# Change in the cause of inpatient mortality after arthroplasty: a retrospective study

**DOI:** 10.1186/s13018-019-1230-x

**Published:** 2019-06-17

**Authors:** Yuzhi Zuo, Jin Lin, Jin Jin, Wenwei Qian, Guixing Qiu, Xisheng Weng

**Affiliations:** Department of Orthopedic Surgery, Peking Union Medical College Hospital, Peking Union Medical College and Chinese Academy of Medical Sciences, No.1 Shuaifuyuan, Beijing, 100730 China

**Keywords:** Joint arthroplasty, Inpatient mortality, Pneumonia, Cardiovascular disease

## Abstract

**Background:**

Although arthroplasty has been proved to be a safe and effective procedure, data regarding inpatient mortality rates associated with arthroplasty in China is unclear. We aimed to investigate the inpatient mortality rate after arthroplasty and the determinants of mortality at our center to ensure improved perioperative management.

**Methods:**

This retrospective study included all patients who underwent arthroplasty at our center. Clinical data of mortality patients were collected. The incidence and the causes of inpatient mortality after arthroplasty were analyzed.

**Results:**

A total of 4176 total knee arthroplasties, 2164 total hip arthroplasties, and 1031 femoral head replacements were performed. A rapid growth in surgery volume was observed, and more than 50% of the surgeries were performed in the last 5 years. The overall inpatient mortality rate is 0.3%; however, the mortality rate even decreased in the last 5 years. The cause of death changed over time. Pneumonia has become the leading cause of death in the past 5 years instead of cardiovascular complications.

**Conclusions:**

Arthroplasty is a safe and effective procedure associated with a relatively low inpatient mortality in China. And inpatient mortality does not increase as the growing surgery volume due to improvement of perioperative management. However, patients presenting with risk factors and those undergoing non-elective procedures demonstrated a relatively high incidence of postoperative complications, particularly pneumonia.

## Background

Arthroplasty including total hip/knee arthroplasty (THA/TKA) and femoral head replacement (FHR) is safe and effective to treat patients with arthropathy like severe osteoarthritis, rheumatoid arthritis, and ankylosing spondylitis, as well as patients presenting with femoral neck fracture. It can significantly relieve the joint pain, restore the joint function, and improve the quality of life [1]. Over the last few decades, advances in surgical technology, prosthetic materials, enhanced recovery therapy strategy, and perioperative management have led to a significant reduction in surgical mortality rates. Owing to advances in the medical field and a rapidly growing aging population, indications of the arthroplasty have broadened. As older patients and those with more comorbidities are undergoing arthroplasties [2], the postoperative complications especially mortality rate remains a serious concern. Reportedly, the inpatient, the 30-day, and the 90- day mortality rates after elective TKA are 0.09–0.13%, 0.2–0.3%, and 0.4–0.7%, respectively; whereas those after elective THA are 0.18–0.19%, 0.2–0.95%, and 0.5–0.7% [3–7], respectively. Fractures increase the risk of postoperative death and the 90-day mortality rate following THA or FHR performed to treat femoral neck fracture is approximately 9.3% [8].

To our knowledge, data regarding inpatient mortality rates associated with arthroplasty in China is unclear. We aimed to investigate the inpatient mortality rate after arthroplasty and to investigate the determinants of mortality at our center to ensure improved perioperative management.

## Methods

This retrospective study included all patients who underwent arthroplasty at our center. The incidence and the causes of inpatient mortality after arthroplasty were analyzed. Clinical data of mortality patients including demographic characteristics, preoperative diagnosis, co-morbidities, American Society of Anesthesiologists (ASA) classification, anesthesiologic method, surgery, postoperative complications, cause of death, and hospital length of stay (LOS) were collected. This study was approved by the Ethics Committee of our hospital.

## Results

**Fig. 1:**
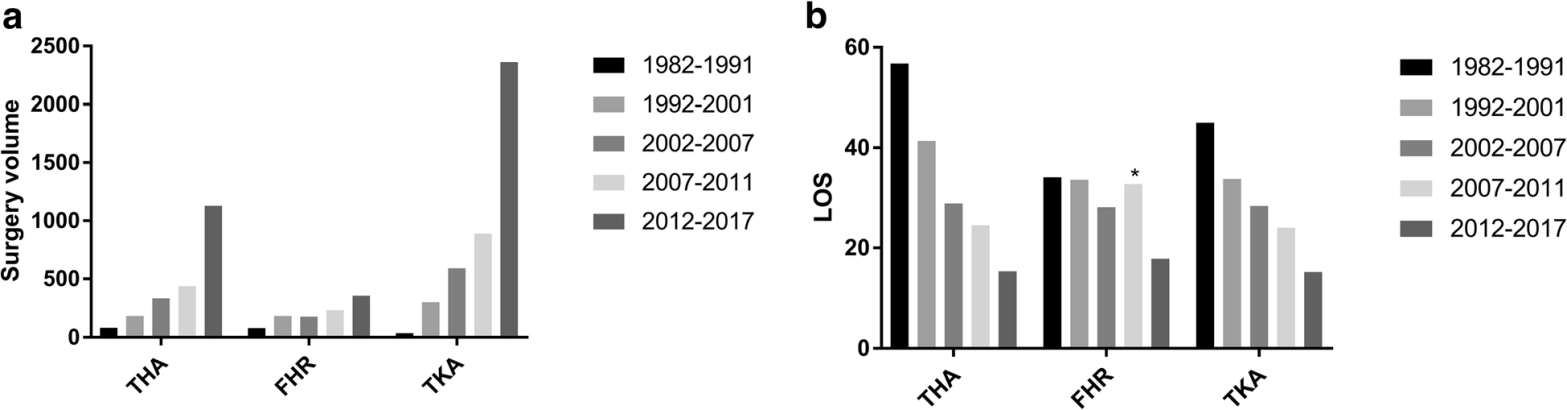
**a**, **b** Trend of surgery volume and hospital length of stay over the last decades. Surgery volume increased rapidly and the LOS decreased significantly especially in the last 5 years. The asterisk indicates there were two patients who stay in hospital for a very long time (685 days and 1083 days) after FHR because of complications during 2007–2011 period. The LOS is shorter than 2002–2007 period after excluding these two patients

A total of 4176 TKAs, 2164 THAs, and 1031 FHRs were performed at our center between 1982 and 2017. Among them, 4054 were elective primary TKAs, 1635 were elective primary THAs, 65 were elective FHRs, and 1194 THAs/FHRs were performed to treat femoral neck fracture. A rapid growth in surgery volume was observed and more than 50% of the surgeries were performed in the last 5 years, with a shorter LOS (Fig. 1).

Overall, 24 patients died during hospitalization owing to postoperative complications after arthroplasty with an overall inpatient mortality rate of 0.3% (24/7371). Four patients died after elective TKA with an inpatient mortality rate of approximately 0.1% (4/4054), and 1 patient died after elective THA with an inpatient mortality rate of approximately 0.06% (1/1635). The remaining patients died after THA/FHR secondary to femoral neck fracture, with an inpatient mortality rate of approximately 1.6% (19/1194). Medical records were missing in 4 patients (two elective TKAs, one elective THA, and one FHR after femoral neck fracture); thus, we analyzed the data obtained from the remaining 20 patients (2 elective and 18 non-elective patients) (Table 1).

**Table 1:** Demographics of mortality patients after joint arthroplasty

	TKA (*n* = 2)	THA/FHR (*n* = 18)	Total (*n* = 20)
Age (year, mean ± SD)	75 ± 4.24	82.28 ± 9.59	81.55 ± .39
Gender (male/female)	0/2	7/11	7/13
Preoperative diagnosis			
Osteoarthritis	2	0	2
Femoral neck fracture	/	18	18
Comorbidity			
Cardiovascular system			
Hypertension	2	9	11
CHD^a^	0	4	4
Old MI^a^	0	2	2
Heart failure	0	1	1
Arrhythmia	1	5	6
Cerebrovascular system			
Old CI^a^	1	4	5
TIA^a^	0	1	1
Perivascular disease			
Arterial atherosclerosis	1	0	1
Venous thrombosis	0	1	1
Pulmonary disease			
COPD^a^	0	0	0
Pulmonary fibrosis	0	1	1
Pneumonia	0	4	4
Respiratory failure	0	1	1
Others			
CKD^a^	0	4	4
Diabetes	1	4	5
Local skin infection	0	2	2
ASA classification			
1–2	2	11	13
3–4	0	7	7
Anesthesia			
GA^a^	1	6	7
CEA/RBA^a^	1	12	13
LOS (day, median (IQR^a^))	22 ± 5.7^b^	26.5 (13.5–81.75)	26 (14.5–71.25)

^a^*CHD* coronary heart disease, *MI* myocardial infarction, *CI* cerebral infarction, *TIA* Transient ischemic attack, *COPD* chronic obstructive pulmonary disease, *CKD* chronic kidney disease, *GA* general anesthesia, *CEA* continuous epidural anesthesia, *RBA* regional block anesthesia, *IQR* interquartile range

^b^Data shows in mean ± SD, because of only two patients in this group

Both patients who died after the elective procedures died of cerebral infarction (CI). One had a history of stroke, and the other had a history of atrial fibrillation. These patients developed CI intraoperatively and on post operation day (POD) 1, respectively.

The 18 patients who underwent non-elective procedures developed 53 postoperative complications including cardiovascular disease, pneumonia, cerebrovascular disease, septic shock, and others such as acute renal failure and local wound infection (Table 2). Among them, pneumonia and cardiovascular disease were the most lethal complications. The cause of death and inpatient mortality rate changed over time. Prior to 2012, cardiovascular complications were the main causes of death; however, after 2012, pneumonia was the leading cause of death (Table 3).

**Table 2:** Postoperative complications in mortality patients after joint arthroplasty

	Elective surgery^b^	Non-elective surgery^b^
	TKA (*n* = 2)	THA/FHR (*n* = 18)
Systemic complications		
Cerebral infarction	2 (100%)	2 (11%)
Acute MI	0	6 (33%)
Arrhythmia	1 (50%)	2 (11%)
Acute heart failure	0	3 (17%)
PE	0	4 (22%)
DVT^a^	0	2 (11%)
Septic shock	0	7 (39%)
Pneumonia	0	10 (56%)
GI^a^ infection	0	3 (17%)
Intracranial infection	0	2 (11%)
Urinary tract infection	0	4 (22%)
Acute renal failure	0	2 (11%)
Bleeding	0	3 (17%)
Local complications		
Deep wound infection	0	1 (6%)
Superficial wound infection	0	1 (6%)
Wound dehiscence	0	1 (6%)

^a^*DVT* deep venous thrombosis, *GI* gastrointestinal

^b^In our study, there are no patients died in hospital after elective THA/FHR or non-elective TKA

**Table 3:** distribution of causes of death of inpatient mortality after joint arthroplasty

Cause of death	Incidence rate							
	1982–1991		1992–2001		2002–2011		2012–2017	
	Elective	Non-elective	Elective	Non-elective	Elective	Non-elective	Elective	Non-elective
Any cause	0	2	0	1	2	9	0	6
MI	0	0	0	1 (100%)	0	2 (22%)	0	0
Arrhythmia	0	1 (50%)	0	0	0	0	0	1 (17%)
PE	0	0	0	0	0	1 (11%)	0	0
CI	0	1 (50%)	0	0	2 (100%)	0	0	0
Pneumonia	0	0	0	0	0	2 (22%)	0	4 (67%)
Abdominal infection	0	0	0	0	0	1 (11%)	0	0
Respiratory failure	0	0	0	0	0	0	0	1 (17%)
MODS^a^	0	0	0	0	0	1 (11%)	0	0
Unclear	0	0	0	0	0	2 (22%)^b^	0	0
All-cause mortality	0 (0/136)	3% (2/65)	0 (0/468)	0.5%(1/198)	0.1% (2/2153)	1.8%(9/505)	0 (0/3420)	1.5%(6/426)

^a^*MODS* multiple organ dysfunction

^b^These two patients died from a sudden breath and cardiac arrest, who probably developed PE or AMI

Seven patients died of postoperative septic shock. Six of them were diagnosed with pneumonia and 1 with abdominal infection. Four of these patients died within the last 5 years. The mean age of patients with pneumonia was 85. Although no patient reported a history of smoking, 3 patients reported preoperative fever and cough. These symptoms were relieved preoperatively following the administration of antibiotics; however, patients with pneumonia deteriorated postoperatively. The other 3 patients with pneumonia were asymptomatic but showed risk factors for pneumonia. One patient presented with delirium and drowsiness preoperatively and could not cooperate with coughing and pulmonary function exercises. He developed pneumonia on POD 1. The second patient developed heart failure, bleeding, and renal failure postoperatively, which led to prolonged immobility. He developed pneumonia after he was admitted to the intensive care unit (ICU). The third patient was a 91-year-old woman who developed pneumonia on POD 13. The patient diagnosed with abdominal infection developed infectious diarrhea secondary to food contamination and showed diffuse peritonitis postoperatively.

Eight patients died of postoperative cardiovascular complications (only 1 patient died in the last 5 years). Three patients presented with acute myocardial infarction (AMI). One reported a history of coronary heart disease (CHD) and was diagnosed as AMI on POD 1, whereas the other 2 denied a history of CHD but reported a history of hypertension and diabetes and were diagnosed with AMI on POD 6 and 7, respectively. Two patients showed severe arrhythmia. One had a history of sinus arrhythmia and developed sudden cardiac arrest intraoperatively, whereas the other had a history of hypertension, CHD, old myocardial infarction, and cardiac dysfunction and died of sudden ventricular fibrillation on POD 60 after developing AMI, heart failure, and pneumonia in quick succession. One patient was diagnosed with pulmonary embolism (PE). He developed sudden dyspnea and chest tightness on POD 14 and the diagnosis was confirmed by computed tomographic pulmonary angiography (CTPA). In addition to the aforementioned patients, 2 patients died of sudden dyspnea or cardiac arrest with a suspected diagnosis of PE or AMI.

Only one 82-year-old woman with a history of stroke, CHD, hypertension, and diabetes died of acute CI on POD 2.

Two patients died of other postoperative complications. One with severe respiratory failure caused by worsening pulmonary fibrosis and the other with multiple organ dysfunction syndrome after developing acute CI, PE, heart failure, renal failure, pneumonia, and gastrointestinal bleeding postoperatively.

## Discussion

To our knowledge, this is the first study to report inpatient mortality rate among Chinese patients undergoing arthroplasty. The overall post-arthroplasty inpatient mortality rate at our center is extremely low, particularly over the last 5 years, which confirms the safety of arthroplasty. However, the mortality rate continues to remain relatively high among those undergoing non-elective procedures.

The cause of death after arthroplasty has changed over time. Previously, cardiovascular disorders including AMI and PE were common in these patients. However, pneumonia has become the most common cause of death over the last 5 years. The perioperative administration of aspirin and low molecular weight heparin has contributed to a reduced incidence of cardiovascular complications.

Owing to the broadened surgical indications, improved surgical skills and the development of highly potent broad-spectrum antibiotics, we have become much more radical to trauma patients with respiratory comorbidities, which has increased the incidence of postoperative pneumonia. Pneumonia is one of the most severe postoperative complications following arthroplasty. The incidence of pneumonia is approximately 0.3%. The mortality risk in patients with postoperative pneumonia is approximately 30-fold higher than that in patients without postoperative pneumonia. Advanced age (especially ≥ 80 years), smoking, chronic obstructive pulmonary disease (COPD), diabetes, hypertension, and other pulmonary diseases are risk factors for postoperative pneumonia [9]. In our study, 50% of patients demonstrated symptoms of pneumonia preoperatively with rapid deterioration in symptoms on POD 1 or 2. However, patients with only risk factors for pneumonia tended to show a later onset of this complication. Therefore, it is recommended that patients with preoperative pneumonia should receive treatment for complete clearance of the infection preoperatively if possible and intra- and postoperative antibiotics should be administered after sensitivity testing. Patients who show risk factors for pneumonia or other postoperative complications caused immobilization should be treated with a prevention program including oral hygiene care, pulmonary function exercises, early mobility practice, head-of-bed elevation to at least 30°, and sitting up for all meals.

Cardiovascular complications including AMI, heart failure, arrhythmia, and PE are the most common cause of postoperative death after arthroplasty [10]. Bass et al. reported that 13.5% of patients aged more than 65 years showed elevation serum levels of high-sensitivity cardiac troponin indicating postoperative myocardial ischemia [11]. However, reportedly, the incidence of symptomatic MI was only 0.2–0.7% [12, 13]. Thus, a considerable number of patients may show transient asymptomatic myocardial ischemia postoperatively. Advanced age, male sex, a history of cardiac disease (CHD, congestive heart failure, valvular disease), pulmonary circulation disorders, vascular disorders, coagulopathy, diabetes, and anemia as well as fluid and/or electrolyte imbalances are independent risk factors for AMI [14]. The inpatient mortality rate in patients with AMI is approximately 3.4%, which is nearly 40-fold higher than that in patients without AMI [15]. In our study, all instances of fatal AMI occurred within the first postoperative week, and they occurred even earlier among patients with a history of CHD. Therefore, we emphasized that patients with risk factors should be closely monitored for the symptoms of myocardial ischemia and dynamic electrocardiogram change. Additionally, assessment of myocardial enzyme is warranted within the first week postoperatively. The incidence of symptomatic deep venous thrombosis (DVT) after arthroplasty was 2.1–12.5% [16, 17]. Without active treatment, approximately 50% of patients are likely to develop PE, and the condition could be fatal in 5–10% of these patients [18]. Depending upon the different methods of anticoagulant, the incidence of postoperative PE is 0.16–1.51% [19]. Our center implemented a postoperative thromboembolism prophylaxis strategy in 2004, which is routinely used presently. Notably, 66.7% (2/3) of fatal PE occurred in 2003–2004; however, this complication is no longer observed since 2010. Although routine thromboembolism prophylaxis can significantly decrease the incidence of fatal PE, it also increases the risk of postoperative bleeding, hematoma, and periprosthetic infection [20, 21]. Therefore, accurate risk stratification for PE is necessary for decision-making regarding optimal anticoagulant treatment in clinical settings.

The incidence of perioperative stroke is 0.08–0.14% [10, 22]. And the inpatient mortality rate in patients who develop stroke postoperatively is approximately 9% [23]. Advanced age, history of stroke, cardiac valvular disease, peripheral vascular disease, atrial fibrillation, diabetes, and coagulopathy are risk factors for perioperative stroke [22]. Therefore, thorough preoperative risk factors screening is important. Patients presenting with risk factors should undergo close monitoring of blood pressure and large intraoperative fluctuations in blood pressure and/or hypotension should be avoided. Perioperative fluid therapy needs to be optimized to avoid hemoconcentration. In this study, stroke onset occurred in patients within 3 days postoperatively. Therefore, high-risk patients should be closely monitored for early abnormalities in neurological and mental symptoms including delay recovery from anesthesia, a disturbance of consciousness, unexplained unilateral extremities immobility and indifference, euphoria, and/or other psychiatric symptoms. Early recognition, early diagnosis, and prompt treatment can prevent the risk of brain injury and decrease the stroke mortality rate.

## Conclusions

This is the first study to analyze the inpatient mortality rates in Chinese patients undergoing arthroplasty. We conclude that arthroplasty is a safe and effective procedure associated with a relatively low inpatient mortality in China. And inpatient mortality does not increase as the growing surgery volume due to improvement of perioperative management. However, patients presenting with risk factors and those undergoing non-elective procedures demonstrated a relatively high incidence of postoperative complications, particularly pneumonia. Thorough preoperative assessment, close perioperative monitoring and the institution of optimal individualized prophylaxis are warranted to reduce inpatient mortality.

## Data Availability

All data generated or analyzed during this study are included in this published article.

## Abbreviations

AMI: Acute myocardial infarction
ASA: American Society of Anesthesiologists
CEA: Continuous epidural anesthesia
CHD: Coronary heart disease
CI: Cerebral infarction
CKD: Chronic kidney disease
COPD: Chronic obstructive pulmonary disease
CTPA: Computed tomographic pulmonary angiography
DVT: Deep venous thrombosis
FHR: Femoral head replacement
GA: General anesthesia
GI: Gastrointestinal
ICU: Intensive care unit
IQR: Interquartile range
LOS: Length of stay
MODS: Multiple organ dysfunction
PE: Pulmonary embolism
POD: Post operation day
RBA: Regional block anesthesia
THA: Total hip arthroplasty
TIA: Transient ischemic attack
TKA: Total knee arthroplasty
